# Effectiveness and safety analysis of SanHanHuaShi granules for the treatment of coronavirus disease 2019: Study protocol and statistical analysis plan for a randomized, parallel-controlled, open-label clinical trial

**DOI:** 10.3389/fphar.2022.936925

**Published:** 2022-08-16

**Authors:** Yangyang Liu, Xi Chen, Hongan Wang, Chensi Yao, Xiaowen Gou, Zezheng Gao, Linlin Sun, Dan Liu, Cheng Tang, Yu Wei, Qiyou Ding, Haoyu Yang, Jiaran Lin, Keyu Chen, Jia Chen, Linhua Zhao, Min Li, Lin Han, Jian Wang, Jixiang Ren, Ying Zhang

**Affiliations:** ^1^ Institute of Integrated Traditional Chinese and Western Medicine, Beijing University of Chinese Medicine, Beijing, China; ^2^ The Affiliated Hospital to Changchun University of Chinese Medicine, Changchun, China; ^3^ Department of Endocrinology, Guang’anmen Hospital of China, Academy of Chinese Medical Sciences, Beijing, China; ^4^ Graduate College, Beijing University of Traditional Chinese Medicine, Beijing, China; ^5^ Gansu University of Chinese Medicine, Lanzhou, China; ^6^ Institute of Metabolic Diseases, Guang’anmen Hospital of China, Academy of Chinese Medical Sciences, Beijing, China; ^7^ Molecular Biology Laboratory, Guang’anmen Hospital of China Academy of Chinese Medical Sciences, Beijing, China; ^8^ School of Traditional Chinese Medicine, Beijing University of Chinese Medicine, Beijing, China

**Keywords:** SanHanHuaShi Granules, Traditional Chinese Medicine, COVID-19, effectiveness, protocol, Statistical analysis plan

## Abstract

**Background:** Coronavirus disease 2019 (COVID-19) was declared a global pandemic in March 2020 by the World Health Organization (WHO). As of July 2, 2022, COVID-19 has caused more than 545 million infections and 6.3 million deaths worldwide, posing a significant threat to human health. Currently, there is still a lack of effective prevention and control strategies for the variation and transmission of SARS-CoV-2. Traditional Chinese medicine (TCM), which has a unique theoretical system, has treated various conditions for thousands of years. Importantly, recent studies have revealed that TCM contributed significantly to COVID-19. SanHanHuaShi (SHHS) granules, a Chinese herbal medicine, which has been included in Protocol for the Diagnosis and Treatment of Novel Coronavirus Disease 2019 (6th to 9th editions) issued by the National Health Commission of China and used to prevent and treat COVID-19 disease. A previous retrospective cohort study showed that SHHS could significantly reduce the severity of mild and moderate COVID-19. However, there is an absence of high-quality randomized controlled clinical studies to confirm the clinical effectiveness of SHHS. Therefore, a clinical study protocol and a statistical analysis plan were designed to investigate the efficacy and safety of SHHS for the prevention and treatment of COVID-19. This study will increase the integrity and data transparency of the clinical research process, which is of great significance for improving the practical application of SHHS granules in the future.

**Methods and analysis:** The study was designed as a 7-day, randomized, parallel controlled, open-label, noninferiority clinical trial of positive drugs. A total of 240 patients with mild and moderate COVID-19 will be enrolled and randomly assigned to receive SanHanHuaShi granules or LianHuaQingWen granules treatment in a 1:1 ratio. Disease classification, vital signs, SARS-CoV-2 nucleic acid testing, symptoms, medications, adverse events, and safety evaluations will be recorded at each visit. The primary outcome will be the clinical symptom recovery rate. Secondary outcomes will include the recovery time of clinical symptoms, negative conversion time of SARS-CoV-2 nucleic acid test negative conversion rate, hospitalization time, antipyretic time, rate of conversion to severe patients, and time and rate of single symptom recovery. Adverse incidents and safety assessments will be documented. All data will be analyzed using a predetermined statistical analysis plan, including our method for imputation of missing data, primary and secondary outcome analyses, and safety outcomes.

**Discussion:** The results of this study will provide robust evidence to confirm the effectiveness and safety of SHHS in the treatment of COVID-19.

**Clinical Trial Registration:** http://www.chictr.org.cn. Trial number: ChiCTR2200058080. Registered on 29 March 2022.

## Introduction

The extremely contagious coronavirus disease 2019 (COVID-19), caused by the severe acute respiratory syndrome coronavirus 2 (SARS-COV-2), has become a global public health concern. COVID-19 has a wide range of clinical manifestations, leading to dysfunction of multiple organs and systems throughout the body and even death (Harrison et al., 2020). The most common symptoms in COVID-19 patients include fever, cough, shortness of breath, fatigue, gastrointestinal problems, and taste and smell disorders (Wiersinga et al., 2020; To et al., 2021). Although people of all ages are vulnerable to infection with the highly transmissible SARS-COV-2, elderly patients or patients with comorbidities are more likely to develop severe or critical disease (Hu B et al., 2021). Therefore, early diagnosis and intervention are essential to control the onset and management of COVID-19. However, currently there is no specific drug with clear evidence of targeting SARS-CoV-2. Therefore, exploring effective prevention and control strategies or drugs against SARS-COV-2 remains an international concern.

The safety and effectiveness of traditional Chinese medicine (TCM) have been widely recognized and evaluated for thousands of years. In the early stages of the outbreak, TCM was incorporated into prevention and treatment strategies, and played an important role in the fight against COVID-19 not only in China but also internationally (Huang et al., 2020; Chu et al., 2021; Huang et al., 2021; Lyu et al., 2021; Wang and Yang, 2021). Owing to the synergistic effect of multiple components and multiple targets, Chinese herbal medicine has a comprehensive therapeutic effect in antiviral, anti-inflammatory, and immunomodulatory functions, thereby aiding in preventing and treating COVID-19 (Li P et al., 2021; Li BH et al., 2021; Ren et al., 2021). With the increase in the number of recovered patients from COVID-19, post-acute sequelae of SARS-CoV-2 infection (PASC) become an important public health problem that can have a long-term impact on pulmonary and multiple extrapulmonary tissues and organs (Wang and Yang, 1827). TCM has a potential advantage in preventing and improving PASC in patients with convalescent COVID-19 (Li et al., 2020; An et al., 2021; Chen et al., 2022).

“Three Chinese medicines and three Chinese recipes” have been included in the 2019 Protocol for Diagnosis and Treatment of Novel Coronavirus Disease 2019 issued by the National Health Commission (NHC) of China and are recommended for the treatment of COVID-19. Several clinical and mechanistic studies have shown that a variety of TCM herbal preparations have the potential to treat COVID-19 (Shen and Yin, 2021; Wu and Zhong, 2021; Yang et al., 2021). LianHuaQingWen (LHQW) capsules or granules have been approved by the NHC to treat COVID-19 and are used extensively worldwide. Multiple randomized controlled clinical trials and mechanistic studies have confirmed the potential advantages of LHQW in improving clinical symptoms and prognosis in patients with COVID-19 (Runfeng et al., 2020; Xiao et al., 2020; Hu K et al., 2021).

Similarly, the Hanshiyi formula recommended in the 2019 Protocol for the Diagnosis and Treatment of Novel Coronavirus Disease 2019 (6^th^ to 9^th^ editions) for patients with mild and moderate COVID-19 is also clinically effective prescription used in many regions of China. To further promote the study and utilize TCM against COVID-19, the Hanshiyi formula was produced as SanHanHuaShi (SHHS) granules after verification of the production process and improvement of quality standards by Jiangsu Kanion Pharmaceutical Co., Ltd. The composition and dosage of the SanHanHuaShi granules were the same as those of the Hanshiyi formula. SHHS is composed of 20 Chinese herbs, which play a role in releasing the exterior and dissipating cold, ventilating the lung and expelling pathogens, dispelling filth, and eliminating turbidity based on the theory of TCM (Han et al., 2020).

Through modern pharmaceutical technology, the Chinese medicine decoction is extracted, concentrated, dried and shaped into a granular formulation. The quality control analysis of SHHS granules utilizing HPLC is provided by Jiangsu Kanion Pharmaceutical Co., Ltd. The quality control data and preliminary identification of the main components are available in **Additional file 1**. The daily dosage of SHHS granules is as follows: Houpo (*Magnolia officinalis* Rehd.et Wils.): 3.13g (equally 15 g herb medicine, abbreviated as 15 g), Jiaobinglang (*Areca catechu* L.):1.88g (9g), Caoguo (*Amomum tsao-ko* Crevost et Lemaire): 9g (1.88g), Mahuang (*Ephedra sinica* Stapf):6g (1.25g), Kuxingren (*Prunus armeniaca* L.var.*ansu* Maxim.): 9g (1.88g), Qianghuo (*Notopterygium incisum* Ting ex H.T.Chang): 15g (3.13g), Shengjiang (*Zingiber officinale* Rosc*.*): 15g (3.13g), Guanghuoxiang (*Pogostemon cablin*(Blanco) Benth.): 15g (3.13g), Peilan (*Eupatorium fortunei* Turcz*.*): 9g (1.88g), Cangzhu (*Atractylodes chinensis*(DC.) Koidz.): 15g (3.13g), Fuling (*Poria cocos*(Schw.)Wolf): 45g (9.38g), Baizhu (*Atractylodes macrocephala* Koidz.**):** 30g (6.25g), Shigao (*Gypsum Fibrosum*): 15g (3.13g), Jiaoshanzha (*Crataegus pinnatifida* Bge.var.*major* N.E.Br.): 9g (1.88g), Jiaomaiya (*Hordeum vulgare* L.): 9g (1.88g), Jiaoshenqu (*Massa Medicata Fermentata*): 15g (3.13g), Dilong (*Pheretima aspergillum* (E.Perrier)): 15g (3.13g), Xuchangqing (*Cynanchum paniculatum*(Bge.)Kitag.): 15g (3.13g), Mianmaguanzhong (*Dryopteris crassirhizoma* Nakai), 9g (1.88g), Tinglizi (*Descurainia sophia*(L.)Webb.ex Prantl.):15g (3.13g).

A previous retrospective cohort study showed that SHHS could considerably reduce the incidence of mild and moderate COVID-19 turning into severe disease (Tian et al., 2020). Although this study suggests that SHHS can prevent and treat COVID-19, there are no further randomized controlled clinical trials to confirm this. Therefore, we hypothesized that SHHS plays a therapeutic role no less than LHQW in patients with mild and moderate COVID-19. Consequently, we designed a randomized, parallel-controlled, open-label clinical trial to demonstrate the efficacy and safety of SHHS in the treatment of COVID-19. The primary objective of this study research is to describe a precise clinical study protocol and a predetermined statistical analysis plan.

## Methods

### Study design

**FIGURE 1:**
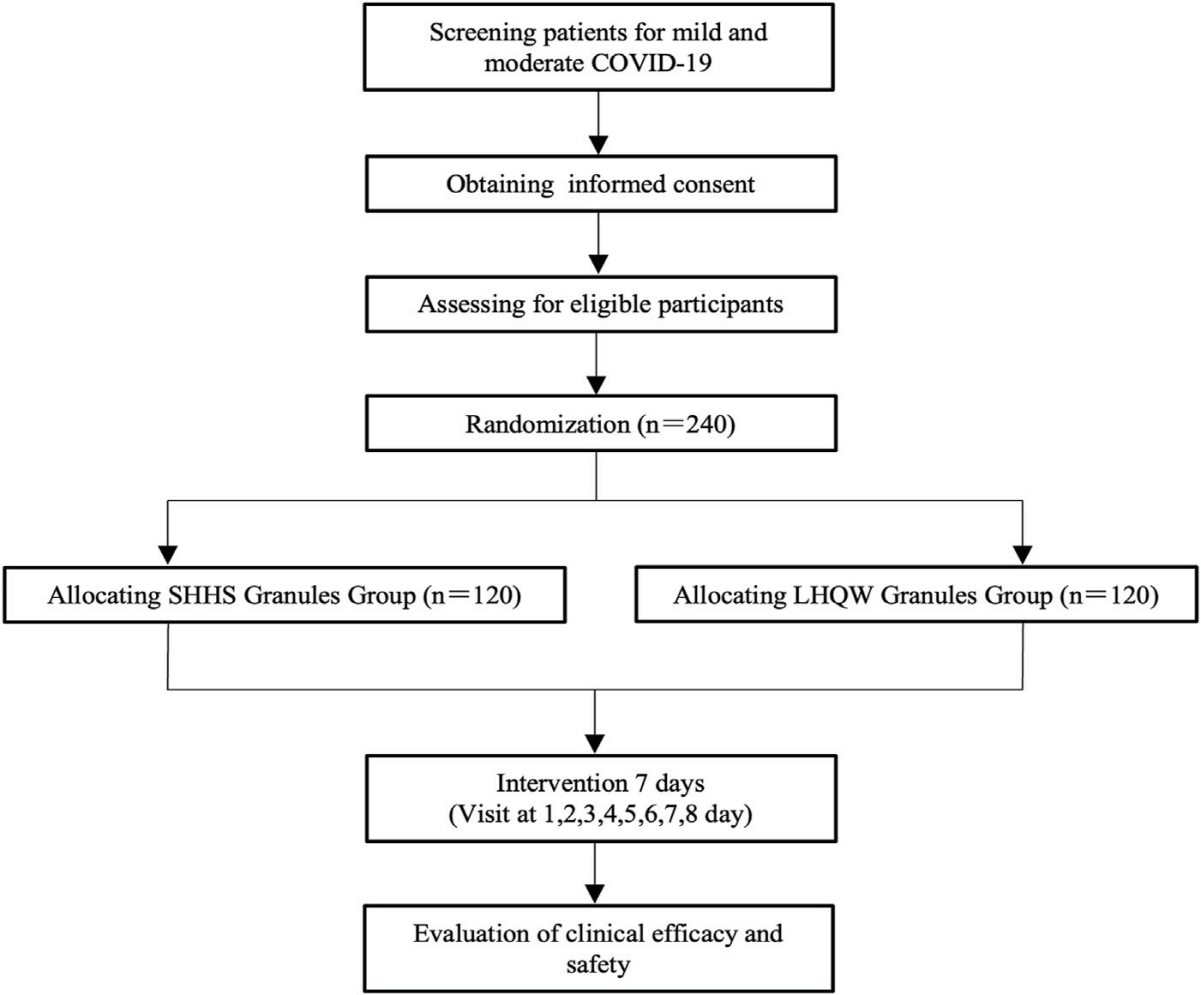
Flow diagram of the study.

This study is a randomized, active parallel-controlled, open-label, and non-inferior phase 2 clinical trial in patients with mild and moderate COVID-19. A total of 240 patients who tested positive for SARS-CoV-2 will be enrolled in this study. The patients will be divided into two randomized groups and treated with granules of either SanHanHuaShi or LianHuaQingWen for 7 days. The Standard Protocol Items: Recommendations for Interventional Trials (SPIRIT) guidelines will be followed (Chan et al., 2013). (**Additional file 2**). The study procedure is summarized in Figure 1. The details of the study schedule for enrollment, interventions, and assessments are presented in Table 1.

**TABLE 1:** SPIRIT Schedule of enrollment, interventions and assessments.

Visit project	Study period									
	Screen period (baseline)	Allocation	Visit							
Time of visit (day)	-1	-1-0	1	2	3	4	5	6	7	8
1. Enrollment										
Eligibility screening	●									
Informed consent	●									
Demographic characteristics	●									
Diagnosis and medical history	●									
Comorbid conditions	●									
2. Interventions										
Random allocation		●								
Administration of study drugs			●	●	●	●	●	●	●	
3. Assessments										
Vital signs	●		●	●	●	●	●	●	●	●
Symptoms	●		●	●	●	●	●	●	●	●
Clinical assessment	●		●	●	●	●	●	●	●	●
Combined drugs	●		●	●	●	●	●	●	●	●
SARS-CoV-2 PCR testing	●		●	●	●	●	●	●	●	●
Adverse event monitoring**			●	●	●	●	●	●	●	●
4. Other work										
Distribute drugs			●	●	●	●	●	●	●	
Recover drugs										●
End of study summary										●

** Throughout the study, ongoing observations and recordings of when this occurred were made.

### Recruitment of participants

This study will include 240 patients who test positive for COVID-19. The patients will be recruited from the Affiliated Hospital of Changchun University of Traditional Chinese Medicine (Jilin province, China). Recruiting advertising will be displayed online and on hospital notice boards. It will contain the study objectives, drugs, medical examinations, and the study eligibility conditions and application method. The study is open to individuals who meet the eligibility criteria and agree to participate. To obtain consent from prospective patients, investigators must outline the study objectives, requirements, and interventions and guarantee their understanding. Two resident physicians will evaluate the patients, each of whom will verify the participants’ study eligibility. Demographic data and reasons for non-participation of ineligible patients will also be documented. The recruitment process started in March 2022 and will last until March 2024. Patients will not participate in study design, recruitment, research procedures, analysis, or dissemination of findings. Medical examinations, laboratory tests, and therapies are provided at no cost to participants, and research may not reimburse them.

### Inclusion criteria

Diagnostic criteria for COVID-19 are based on the Protocol for the Diagnosis and Treatment of Novel Coronavirus Disease 2019 (9th edition) released by the NHC of the People's Republic of China (General Office Of The National Health And Health Commission, 2022) and include individuals who qualified for eligibility meeting the following criteria: 1) clinically confirmed patients of COVID-19 according to the Protocol for Diagnosis and Treatment of Novel Coronavirus Pneumonia (9th edition) with laboratory confirmed SARS-CoV-2 infection as determined by RT-PCR; 2) patients judged to have mild or moderate COVID-19. (Patients with mild COVID-19 usually present with mild clinical symptoms, without radiological manifestations of pneumonia, whereas patients with moderate COVID-19 patients have a fever and respiratory symptoms, as well as radiological features of pneumonia); 3) presence of one or more symptoms of fever, cough, sore throat, and fatigue when entering the group; 4) the time of admission is not more than 24 hours; 5) patients of either sex who are 18 years or older, and 6) providing written informed consent before enrolling in the trial.

### Exclusion criteria

Patients are ineligible for the study if they meet any of the following criteria: 1) show the early warning indicators of severe or critical condition; 2) use similar Chinese patent drugs before enrollment; 3) immunodeficiency diseases or use of immunosuppressants or glucocorticoids within the last 3 months; 4) pregnancy, lactation, or intention to become pregnant; 5) allergic constitution (referring to those allergic to more than two types of drugs or foods or the known ingredients of the investigational medications); 6) mental illness, or without self-knowledge ability, and 7) other conditions judged by the investigators.

### Withdrawal criteria and termination criteria

First, the investigator's decision to withdraw the patient is based on the following principles: 1) during the clinical trial, the patients develop diseases that are not suitable for further study; 2) patients with adverse events or serious adverse events are not suitable to continue the study, and 3) other conditions that the researchers believe will harm the health of the patients by continuing the study.

Second, patients can and should withdraw if they are unwilling to continue with the clinical trial. According to the provisions of the informed consent form, participants can withdraw at any time during the study. If patients have not explicitly proposed to withdraw but no longer accept medication and testing, as well as loss to follow-up, are regarded as 'withdrawal'. The reasons for withdrawal should be known and recorded as precisely as possible. Examples include consciously being unable to tolerate adverse events, inability to continue clinical research due to other reasons, or loss to follow-up without explaining the reasons. In electronic case report forms (eCRFs), the reasons for dropout will be recorded and the data analysis will include their last record.

Third, when reviewing the data prior to statistical analysis, it is up to the principal investigator (PI) and statisticians to judge whether an individual case is excluded. If one of the following events occurs, the two sides should consider whether the patient should be excluded based on the study's progress and the reasons for withdrawal and provide a detailed explanation: 1) After randomization, individuals not meeting the selection criteria or meeting the exclusion criteria of the research proposal are found; 2) The failure to take at least one dose of trial medication and the lack of any data post-randomization.

Fourth, the conditions for stopping early are as follows: 1) if a high incidence of serious adverse events is found during the research, the study should be suspended in time; 2) if during the research, significant mistakes are found in the research proposal or there are serious deviations in its implementation, making it difficult to evaluate the efficacy of drugs, the research should be stopped; 3) the ethics committee requests to suspend or terminate the research; 4) the administrative department cancels the research.

### Sample size

The study hypothesis is SHHS group will be non-inferior to LHQW group. Clinicians and statisticians set the non-inferiority threshold at 10% and predicted that the recovery rate of clinical symptoms after 7 days of LHQW is about 87% and that of SHHS is 90% according to the literature reports on similar drugs. It is estimated that 94 patients for each group, ie. a total of 188 patients are needed to achieve the power of 80% at a one-sided statistically significant level of 0.025. Considering the possible dropout rate of 20%, 240 patients will be enrolled, that is 120 patients for each group.

### Randomization and allocation

A permuted block randomization procedure will be used to allocate 240 eligible patients in a 1:1 ratio. Randomization numbers will be generated using the PROC PLAN process statement in the SAS statistical software package (version 9.4; SAS Inc., Cary, NC, USA). Patients will be assigned to either the SHHS or LHQW group. The Department of Statistics at the Nanjing Medical University will be responsible for randomization. Randomization code will be concealed in opaque envelopes and the allocation information will not be known before randomization procedure.

### Interventions measures

Patients in the SHHS group will receive SHHS granules (10 g per bag), two bags at a time, three times a day, to be swallowed with warm water. The administration of LHQW granules (10 g per bag) will be the same as the SHHS group. The medication course was 7 days (the medication could be stopped in advance if the discharge standard was reached). It will be forbidden to receive other TCM for COVID-19 treatment during the treatment period, including TCM decoctions, granules, or proprietary Chinese medicine. The types, dosage, and cumulative time (including the start and end dates) of the combined therapy drugs should be recorded during the observation period. The intervention will be stopped if there are any severe adverse effects or withdrawal.

### Blinding

The SHHS used in this study is provided by Jiangsu Kanion Pharmaceutical Co. Ltd. (Lianyungang City, Jiangsu Province, China). LianHuaQingWen granules were purchased from Shijiazhuang Yiling Pharmaceutical Co., Ltd. (Shijiazhuang, China). All medications are administered under good manufacturing practices. Even though the trial is open-label, the research assistants who allocate patients to groups will be unaware of their treatment allocation before grouping. Also, assessors and statisticians will be blinded to the group assignment when assessing the efficacy and analyzing the data. Before the analysis and evaluation, all samples and data are anonymized.

### Adherence to study protocol

Protocol adherence during the treatment period will be assessed at each visit and will be based on medication adherence. Frequency distributions are used to describe the proportion of patients in both groups. Medication adherence in the two groups will be calculated and compared according to the ratio of the actual drug administered to the recommended dose. The drugs distributed to the patients and those not administered are documented every day.

## Outcome measures

### Baseline measures for eligibility

During the treatment period, CRFs will be completed with information about the demographic and clinical characteristics of the patients, including age, sex, height, weight, and medical and treatment histories.

### Primary outcomes

The primary outcome is the rate of recovery from four types of patients' self-reported clinical symptoms, including fever, coughing, sore throat and fatigue. It specifically refers to the proportion of patients who met the criteria at the baseline for specific clinical symptoms and achieved recovery at the end of treatment on the seventh day.

### Secondary outcomes

The secondary outcomes are the recovery time of patients self-reported clinical symptoms, SARS-CoV-2 nucleic acid testing negative conversion time, and negative conversion rate, hospitalization time, antipyretic time, the incidence of severe patients, single symptom disappearance time, and disappearance rate.

Criteria for judging the curative effect:

(1) Recovery time and cure rate of clinical symptoms: The recovery of clinical symptoms refers to the disappearance of the main symptoms, ie. fever, coughing, sore throat, and fatigue. The cure rate is defined as the proportion of patients whose clinical symptoms recovered after the end of treatment on the seventh day.
(2) SARS-CoV-2 nucleic acid testing negative conversion time and negative conversion rate: Taking the first time of the two consecutive respiratory tract SARS-CoV-2 PCR negative tests (sampling interval at least 1 d) as the SARS-CoV-2 PCR testing negative time (the SARS-CoV-2 nucleic acid testing negative conversion time equals the SARS-CoV-2 PCR testing negative time–the diagnosis time of the patient).
(3) Hospitalization time: the time from patient admission to discharge. The discharge standard refers to the release from isolation or discharge criterion of the protocol for diagnosing and treating novel coronavirus pneumonia (9th edition).
(4) Antipyretic time: The body temperature is less than 37.4°C after treatment, for 24 h or more.
(5) The conversion rate of turning to severe patients: the proportion of patients who develop severe infection 7 days after taking medicine.

### Safety outcomes and adverse events (AEs)

AEs will be continuously checked during the 7-day treatment period. AEs indices will include all adverse events. At any time, all information about adverse events, including their severity, duration, and correlation to SHHS granules, should be recorded in detail. The AEs was graded as mild, moderate, or severe. Mild AEs could be defined as situations that could be tolerated without any further medical therapies. Moderate AEs is pain or discomfort that patients can not tolerate and require medical treatments. Severe AEs means that, after receiving the medications used in the study, the patients have a dead, life-threatening, permanent, or severe disability or loss of function; the patients need hospitalization or extended hospitalization or lead to congenital abnormalities or birth defects. AEs must be reported to the research center, principal sponsor, and research medical ethics committee as soon as possible. At the same time, the National Medicinal Product Administration (NMPA) will be notified within 24 h. A serious adverse event must also be completed as soon as possible.

### Data collection, management, and quality control

An electronic data capture (EDC) system will gather and manage data in this trial. The electronic case report form (eCRF) data matches the information in the original medical records, laboratory examination reports, and other sources. According to the research protocol, the eCRF should guarantee that all the data required for analysis are collected efficiently and precisely. To maintain the quality of the data and guarantee adherence to the protocol, the sponsor and investigators will establish quality control and quality assurance systems, respectively. Investigators and research assistants who participate in clinical research must have professional expertise, qualifications, and abilities in clinical research. They should explicitly explain the protocols for patient selection, data input, medication use, AEs reporting, and dropout documentation. They should conscientiously and patiently explain to the patients to fully understand and cooperate with the research to provide good patient compliance. Patients should be informed of possible adverse reactions to the medication and the coping approach to be taken in the event of adverse reactions.

It is necessary to protect clinical research data's authenticity, integrity, and privacy and to guarantee the traceability of clinical research data. For the questions existing in the data, the data administrator will fill in the concentrator data-ready queue (DRQ) and send a query to the investigators through the clinical monitor. Investigators should answer this question and provide feedback as soon as possible. The data administrator will modify the data according to the investigators' answers, confirm and enter them, and send the DRQ again if necessary. Data verification includes computerized program verification (edit check), manual verification, and data verification meetings. Any inconsistent data (data query) found in the verification shall be corrected in time. The data department shall issue a discrepancy report and submit it to the researcher for confirmation and changes. At the end of the study, the researchers and statistical analysts will review the data and lock them after confirming that they are correct. All electronic data will be stored in password-protected files on designated computers that researchers can access. The necessary documents for this study shall be archived and managed in relevant drug clinical research institutions and statistical units, respectively. The research data shall be kept until five years after the end of the study.

## Statistical analysis plan

### Statistical methods

The statistical analysis plan will comply with the guidelines for the content of statistical analysis plans in clinical trials (Gamble et al., 2017). (**Additional File 3**). After finishing the trial, all statistical analysis tests will be conducted using SAS (version 9.4; SAS Inc., Cary, NC, USA) and one-sided tests with a nominal alpha level of 0.025. All confidence intervals (Cls) will be stated at the 95 confidence level. To assess non-inferiority, the CI lower limit of the difference between groups will be compared with a predefined non-inferiority limit. Non-inferiority could only be declared when the 95% CI lower limit of the difference between groups is not larger than the non-inferiority threshold of 10%.

Continuous outcomes will be described by calculating the mean, standard deviation, median, and minimum and maximum values. The means and standard deviations are reported for normal continuous data. For non-normally distributed continuous variables, the median and interquartile ranges are given. The frequency and proportion of each category are displayed as counting indications.

Between-group comparisons are performed using an independent two-sample t-test (normality, homogeneity of variance) or the Wilcoxon rank-sum test (non-normality, heterogeneity of variance). The paired t-test is used for comparisons within normally distributed groups, and the paired rank-sum test is used for comparisons within non-normally distributed groups. For count metrics, the χ^2^ test or Fisher's exact probability will be required, whereas, for categorical variables, the Wilcoxon rank-sum test will be utilized. Repeated measured outcomes are applied to a generalized linear mixed model analysis.

In patients where baseline indicators are unbalanced, an adjustment to the efficacy contrast of variables such as age, sex, and concurrent therapy is required. The covariance approach is employed for continuous variables, and for count or rank data, logistic regression analysis is performed.

### Definition of analysis sets

(1) Full analysis set (FAS): All participants randomly assigned to the group, use the medicine at least once, and have at least one visit will be analyzed using intention-to-treat analysis of the entire dataset.
(2) Per protocol set (PPS): This includes all participants who comply with the research protocol, which have no missing data on important baseline variables and primary outcome. Major violations on concomitant medicine and extremely lower compliance will be considered to be excluded from PPS.
(3) Safety set (SS): All randomized participants could be included in SS if they have used at least one dose of drugs and have completed at least a one-time safety assessment. Impution will not be conducted for safety outcomes in SS.

### Descriptive statistics

Participants' baseline characteristics will be reported in a baseline table for each randomization group, and baseline data and descriptive statistics will be assessed using standard measures in accordance with the recommendations of the consolidated standards of reporting trials (CONSORT) guidelines (Moher et al., 2010). (Table 2). The table describes the following variables: age, sex, height, weight, clinical symptoms, and medical and treatment histories. The sample is displayed in accordance with the entire analysis set. Baseline characteristics will be compared between the groups.

**Table 2:** Baseline demographic and clinical characteristics.

Variables	SHHS Group (n=xx)	LHQW Group (n=xx)	All participants (n=xx)	*P-value*
Age (year)^a^	xx (xx)	xx (xx)	xx (xx)	.xx
18≤Age<65	xx (xx)	xx (xx)	xx (xx)	.xx
Age≥65	xx (xx)	xx (xx)	xx (xx)	.xx
Males sex^b^	xx (xx%)	xx (xx%)	xx (xx%)	.xx
Height (cm)^a^	xx (xx)	xx (xx)	xx (xx)	.xx
Weight (kg)^a^	xx (xx)	xx (xx)	xx (xx)	.xx
Vital signs				
Heart rate (beats/minute)^a^	xx (xx)	xx (xx)	xx (xx)	.xx
Respiratory rate (breathe / minute) a	xx (xx)	xx (xx)	xx (xx)	.xx
Pulse Rate (beats/minute)^a^	xx (xx)	xx (xx)	xx (xx)	.xx
Systolic blood pressure (mmHg)^a^	xx (xx)	xx (xx)	xx (xx)	.xx
Diastolic blood pressure (mmHg)^a^	xx (xx)	xx (xx)	xx (xx)	.xx
Temperature (°C)^a^	xx (xx)	xx (xx)	xx (xx)	.xx
Past medical history^b^	xx (xx%)	xx (xx%)	xx (xx%)	.xx
Diabetes^b^	xx (xx%)	xx (xx%)	xx (xx%)	.xx
High blood pressure^b^	xx (xx%)	xx (xx%)	xx (xx%)	.xx
Coronary artery disease^b^	xx (xx%)	xx (xx%)	xx (xx%)	.xx
Chronic obstructive pulmonary disease^b^	xx (xx%)	xx (xx%)	xx (xx%)	.xx
Stroke^b^	xx (xx%)	xx (xx%)	xx (xx%)	.xx
Cancer^b^	xx (xx%)	xx (xx%)	xx (xx%)	.xx
Heart failure^b^	xx (xx%)	xx (xx%)	xx (xx%)	.xx
···	···	···	···	···
Disease diagnosis^b^	xx (xx%)	xx (xx%)	xx (xx%)	.xx
Mild^b^	xx (xx%)	xx (xx%)	xx (xx%)	.xx
Moderate^b^	xx (xx%)	xx (xx%)	xx (xx%)	.xx
Initial symptoms^b^				
Fever^b^	xx (xx%)	xx (xx%)	xx (xx%)	.xx
Cough^b^	xx (xx%)	xx (xx%)	xx (xx%)	.xx
Fatigue^b^	xx (xx%)	xx (xx%)	xx (xx%)	.xx
Diarrhea^b^	xx (xx%)	xx (xx%)	xx (xx%)	.xx
Shortness of breath^b^	xx (xx%)	xx (xx%)	xx (xx%)	.xx
Sore throat^b^	xx (xx%)	xx (xx%)	xx (xx%)	.xx
Nausea^b^	xx (xx%)	xx (xx%)	xx (xx%)	.xx
Vomiting^b^	xx (xx%)	xx (xx%)	xx (xx%)	.xx
Taste and smell dysfunction^b^	xx (xx%)	xx (xx%)	xx (xx%)	.xx
Loss of appetite^b^	xx (xx%)	xx (xx%)	xx (xx%)	.xx
···	···	···	···	···
Concomitant medications	xx (xx%)	xx (xx%)	xx (xx%)	.xx
Antiviral drugs^b^	xx (xx%)	xx (xx%)	xx (xx%)	.xx
Anti-infective drugs^b^	xx (xx%)	xx (xx%)	xx (xx%)	.xx
Chinese patent medicine^b^	xx (xx%)	xx (xx%)	xx (xx%)	.xx
Others^b^	xx (xx%)	xx (xx%)	xx (xx%)	.xx

a Number, mean, standard deviation;

b Number, percentage.

### Imputation of missing data

Missing values of the primary and secondary outcomes will be carried forward from the latest observation data to the final results (LOCF method).

### Analysis of primary outcome

The primary analysis will be conducted on the FAS and PPS, which is the rate of self-reported clinical symptoms, including fever, fatigue, and coughing recovery. The percentage of patients whose clinical symptoms disappeared on the seventh day will be summarized. CMH-Chisquare test will be applied to compare rates between groups and Clopper-Pearson method will be used to calculate the 95%CI for rate difference (Table 3).

**Table 3:** Analysis of the primary outcome.

	Full analysis set				Per Protocol set			
Variables	SHHS group (n=xx)	LHQW Group (n=xx)	Rate difference 95%CI	*P-Value*	SHHS group (n=xx)	LHQW Group (n=xx)	Rate difference 95%CI	*P-Value*
Rate of clinical symptom recovery at days 7 (n,%)^b^	xx (xx%)	xx (xx%)	xx%(xx% to xx%)	.xx	xx (xx%)	xx (xx%)	xx%(xx% to xx%)	.xx

b Number, percentage.

### Analysis of secondary outcomes

The secondary outcomes of the FAS and PPS will be analyzed, including the recovery time of clinical symptoms, SARS-CoV-2 nucleic acid testing negative conversion time and negative conversion rate, hospitalization time, antipyretic time, the conversion rate of turning to severe patients, disappearance time of single symptoms, and disappearance rate. All secondary outcomes will be compared between and within groups based on the distribution and changes at baseline and each visit point (Table 4).

**Table 4:** Analysis of secondary outcomes.

	Full analysis set				Per Protocol set			
Variables	SHHS group (n=xx)	LHQW Group (n=xx)	Mean difference 95%CI	*P-Value*	SHHS group (n=xx)	LHQW Group (n=xx)	Mean difference 95%CI	*P-Value*
Time to clinical symptom recovery^a^	xx (xx)	xx (xx)	xx(xx to xx)	.xx	xx (xx)	xx (xx)	xx(xx to xx)	.xx
Time to single symptom recovery^a^								
Fever^a^	xx (xx)	xx (xx)	xx(xx to xx)	.xx	xx (xx)	xx (xx)	xx(xx to xx)	.xx
Cough^a^	xx (xx)	xx (xx)	xx(xx to xx)	.xx	xx (xx)	xx (xx)	xx(xx to xx)	.xx
Fatigue^a^	xx (xx)	xx (xx)	xx(xx to xx)	.xx	xx (xx)	xx (xx)	xx(xx to xx)	.xx
Diarrhea^a^	xx (xx)	xx (xx)	xx(xx to xx)	.xx	xx (xx)	xx (xx)	xx(xx to xx)	.xx
Shortness of breath^a^	xx (xx)	xx (xx)	xx(xx to xx)	.xx	xx (xx)	xx (xx)	xx(xx to xx)	.xx
Sore throat^a^	xx (xx)	xx (xx)	xx(xx to xx)	.xx	xx (xx)	xx (xx)	xx(xx to xx)	.xx
Nausea^a^	xx (xx)	xx (xx)	xx(xx to xx)	.xx	xx (xx)	xx (xx)	xx(xx to xx)	.xx
Vomiting^a^	xx (xx)	xx (xx)	xx(xx to xx)	.xx	xx (xx)	xx (xx)	xx(xx to xx)	.xx
Taste and smell dysfunction^a^	xx (xx)	xx (xx)	xx(xx to xx)	.xx	xx (xx)	xx (xx)	xx(xx to xx)	.xx
Loss of appetite^a^	xx (xx)	xx (xx)	xx(xx to xx)	.xx	xx (xx)	xx (xx)	xx(xx to xx)	.xx
···	···	···	···	···	···	···	···	···
Rate of single symptom recovery^b^								
Fever^b^	xx (xx%)	xx (xx%)	xx(xx to xx)	.xx	xx (xx%)	xx (xx%)	xx(xx to xx)	.xx
Cough^b^	xx (xx%)	xx (xx%)	xx(xx to xx)	.xx	xx (xx%)	xx (xx%)	xx(xx to xx)	.xx
Fatigue^b^	xx (xx%)	xx (xx%)	xx(xx to xx)	.xx	xx (xx%)	xx (xx%)	xx(xx to xx)	.xx
Diarrhea^b^	xx (xx%)	xx (xx%)	xx(xx to xx)	.xx	xx (xx%)	xx (xx%)	xx(xx to xx)	.xx
Shortness of breath^b^	xx (xx%)	xx (xx%)	xx(xx to xx)	.xx	xx (xx%)	xx (xx%)	xx(xx to xx)	.xx
Sore throat^b^	xx (xx%)	xx (xx%)	xx(xx to xx)	.xx	xx (xx%)	xx (xx%)	xx(xx to xx)	.xx
Nausea^b^	xx (xx%)	xx (xx%)	xx(xx to xx)	.xx	xx (xx%)	xx (xx%)	xx(xx to xx)	.xx
Vomiting^b^	xx (xx%)	xx (xx%)	xx(xx to xx)	.xx	xx (xx%)	xx (xx%)	xx(xx to xx)	.xx
Taste and smell dysfunction^b^	xx (xx%)	xx (xx%)	xx(xx to xx)	.xx	xx (xx%)	xx (xx%)	xx(xx to xx)	.xx
Loss of appetite^b^	xx (xx%)	xx (xx%)	xx(xx to xx)	.xx	xx (xx%)	xx (xx%)	xx(xx to xx)	.xx
···	···	···	···	···	···	···	···	···
Time to negative conversion of SARS-CoV-2 PCR testing^a^	xx (xx)	xx (xx)	xx(xx to xx)	.xx	xx (xx)	xx (xx)	xx(xx to xx)	.xx
Rate of negative conversion of SARS-CoV-2 PCR testing^b^	xx (xx%)	xx (xx%)	xx(xx to xx)	.xx	xx (xx%)	xx (xx%)	xx(xx to xx)	.xx
Rate of conversion of severe patients^b^	xx (xx%)	xx (xx%)	xx(xx to xx)	.xx	xx (xx%)	xx (xx%)	xx(xx to xx)	.xx
Antipyretic time^a^	xx (xx)	xx (xx)	xx(xx to xx)	.xx	xx (xx)	xx (xx)	xx(xx to xx)	.xx
hospitalization time^a^	xx (xx)	xx (xx)	xx(xx to xx)	.xx	xx (xx)	xx (xx)	xx(xx to xx)	.xx

a Number, mean, standard deviation;

b Number, percentage.

Continuous data will be summarized using number, mean, standard deviation, minimum, maximum, median, upper and lower quartile. A t-test or Wilcoxon rank test will be applied to compare groups. ANCOVA will be considered to evaluate the influence of covariates. Chi-square test or Fisher exact test will be used to compare groups for categorical data. CMH-Chisquare test and logistic regression will be considered to explore the influence of covariates. Time-to-event data will be estimated by Kaplan-Meier method to compute the median and corresponding 95%CI. Log-rank test will be used for between-group comparison.

### Analysis of safety outcomes

All participants in the safety set are examined during safety analysis. All AEs that occurred during the intervention period are recorded, including their type, severity, frequency, and relationship to the intervention medicine. The quantity and frequency of adverse events are calculated for both groups. Severe adverse events and consequent drug discontinuation are meticulously recorded. Safety analysis will use qualitative judgment to define the distribution, counting the number of participants and their fraction of abnormal indicators using cross-tabulation (Table 5). The correlation between AE and medications will be evaluated using standards from the 2011 Ministry of Health of the People's Republic of China's Measures for the Reporting and Monitoring of Adverse Drug Reactions.

**Table 5:** Analysis of the adverse events in the full analysis set.

Adverse event	SHHS Group (n=xx)	LHQW Group (*n*=xx)	*P-Value*
In total^b^	xx(xx%)	xx(xx%)	.xx
Headache^b^	xx(xx%)	xx(xx%)	.xx
Nausea^b^	xx(xx%)	xx(xx%)	.xx
Vomiting^b^	xx(xx%)	xx(xx%)	.xx
Diarrhea^b^	xx(xx%)	xx(xx%)	.xx
Loss of appetite^b^	xx(xx%)	xx(xx%)	.xx

^b^Number, percentage.

### Ethics and dissemination

The study protocol was approved by the Ethics Committee of the Affiliated Hospital of Changchun University of Traditional Chinese Medicine (no. CCZYFYLL2022). All participants will provide written informed consent. The results of this study will be published in peer-reviewed journals.

## Discussion

The ongoing COVID-19 pandemic, which has been spreading for more than two years, has led to various variants of SARS-CoV-2 (Tao et al., 2021; Khandia et al., 2022). The increased transmissibility or severity of SARS-CoV-2 continually poses serious challenges to vaccine development and targeted therapeutic strategies (Dong et al., 2021; Lino et al., 2022). Timely intervention with TCM in COVID-19 has been shown to significantly relieve symptoms, shorten the course, and improve the prognosis of the disease An et al., 2021. The trial is a randomized, active-drug parallel-controlled, open-label, non-inferiority clinical trial carried out in China to evaluate the effectiveness of SHHS compared to LHQW among patients with COVID-19. This phase 2 clinical trial will provide clinical evidence of TCM herbal preparations for preventing and treating COVID-19. The SAP was developed to prepare for statistical analysis of all data after the completion of this study and locking of the database. Although this is a single-center and open-label study, the quality of evidence will be improved by strict quality control and transparent reporting. The findings of this study will have important implications both for Chinese and western clinical practices. Furthermore, if the non-inferior conclusion could be declared in our study, SHHS granules will be confirmed its effects not only for symptoms management of COVID-19 and reducing the severe incidence, but also for extended indications of virus pneumonia or influenza. All of these depend on integrated multi-source high-quality data including randomized controlled trials and observational studies in the future.

## Data Availability

The original contributions presented in the study are included in the article/Supplementary Material further inquiries can be directed to the corresponding authors.
