# Supplementary material for: Effectiveness and safety analysis of SanHanHuaShi granules for the treatment of coronavirus disease 2019: Study protocol and statistical analysis plan for a randomized, parallel-controlled, open-label clinical trial
Source: Front Pharmacol. 2022 Aug 16;13:936925. doi: 10.3389/fphar.2022.936925 (PMC9425051; doi:10.3389/fphar.2022.936925)
Supplement: Supplementary file 3 [file DataSheet1.docx]

**Materials and methods**

1. SHHS granules were manufactured by Jiangsu Kangyuan Pharmaceutical Co., Ltd.

1g SHHS granules were weighed and dissolved by methanol, the solution was passed through a neutral alumina column, eluted with ethanol, and 30ml of the eluate was collected, added with hydrochloric acid, mixed, evaporated to dryness, and dissolved in 50% methanol for further testing. Ephedrline hydrochloride (batch No. 171241-201809, CFDA) and pseudoephedrine hydrochloride (batch No. 171237-201510, CFDA) were dissolved by methanol.

The HPLC analysis of SHHS granules was carried out on a Waters HPLC (Waters e2695+2998). The chromatographic separation was performed using a Waters Symmetry Excel C18 column (250×4.6mm, 5μm) at 25℃, and cetonitrile-0.1% phosphoric acid solution (4:96) was used as the mobile phase for analysis. The flow rate was set at 1.0 mL/min. 10 μL of the sample was injected into the HPLC system for analysis and detected at 210 nm.

**Results**

The HPLC of standards and SHHS are shown in Figure 1 and Figure 2 respectively. The total contents of ephedrine hydrochloride and pseudoephedrine hydrochloride in SHHS granules was 0.33mg-0.98mg/g.

Figure 1. The HPLC profile of the control.

Figure 2: The HPLC chart of SHHS Granules

**Materials and methods**

1. 1g SHHS granules were weighed and dissolved by 50% methanol for further testing. Magnolol (batch No. 110729-202015, CFDA) [and](https://cn.bing.com/dict/search?q=and&FORM=BDVSP6&cc=cn) honokiol ( batch No. 110730-201915, CFDA) were dissolved by 50% methanol. The HPLC analysis of SHHS granules was carried out on a Waters HPLC (Waters e2695+2998). The chromatographic separation was performed using a Waters Symmetry Excel C18 column (250×4.6mm, 5μm) at 25℃, acetonitrile (A), and 0.1% phosphoric acid solution (B) was used as the mobile phase for analysis. The flow rate was set at 1.0 mL/min. The elution conditions were applied using a gradient program as follows: 40-85%a for 0-40 min，85%-40%a for 40-45 min，40%a for 45-55 min, 10 μL of the sample was injected into the HPLC system for analysis and detected at 210 nm.

**Results**

The HPLC of standards and SHHS are shown in Figure 3 and Figure 4 respectively. The total contents of magnolol and honokiol in SHHS granules was 0.087mg-0.290 mg/g.

Figure 3. The HPLC profile of the control.

Figure 4: The HPLC chart of SHHS Granules

**Materials and methods**

1. 0.8g SHHS granules were weighed and dissolved by 50% methanol for further testing. Nodakenin (batch No.111821-201604, CFDA) were dissolved by 50% methanol. The HPLC analysis of SHHS granules was carried out on a Waters HPLC (Waters e2695+2998). The chromatographic separation was performed using a Waters Symmetry Excel C18 column (250×4.6mm, 5μm) at 25℃, methanol-0.1% phosphoric acid (35:65) was used as the mobile phase for analysis. The flow rate was set at 1.0 mL/min. 10μL of the sample was injected into the HPLC system for analysis and detected at 335 nm.

**Results**

The HPLC of standards and SHHS are shown in Figure 5 and Figure 6 respectively.

The content of nodakenin in SHHS granules was 0.56mg-4.09mg/g.

Figure 5. The HPLC profile of the control.

Figure 6: The HPLC chart of SHHS Granules

**Materials and methods**

1. 0.8g SHHS granules were weighed and dissolved by water for further testing. Adenine (batch No.B0002908, Beijing Manhag Biotechnology Co., Ltd. ); uridine (batch No.B0008580, Beijing Manhag Biotechnology Co., Ltd. ); guanosine (batch No.B0006767, Beijing Manhag Biotechnology Co., Ltd. ); inosine ( batch No. 140669-202007, CFDA) ; 5-hydroxymethyl-2-furaldehyde (batch No.ST00720220, Shanghai Shidander Standard Technology Co., Ltd.); magnoloside A (batch No.STC0610110, Shanghai Shidander Standard Technology Co., Ltd.); bergaptol-β-glucopyranoside (batch No.CFS202002, Shanghai Shidander Standard Technology Co., Ltd.); nodakenin ( batch No. 111821-201604, CFDA); 6-Feruloylnodakenin (batch No.CFS202002, Shanghai Shidander Standard Technology Co., Ltd.) ;4-hydroxyphenethylanisate (batch No.A05GB156933, Shanghai Yuanye Biotechnology Co., Ltd.) were dissolved by 50% methanol.

The HPLC analysis of SHHS granules was carried out on a Waters HPLC (Waters e2695+2998). The chromatographic separation was performed using a Waters Symmetry Excel C18 column (250×4.6mm, 5μm) at 25℃, methanol-0.1% phosphoric acid (35:65) was used as the mobile phase for analysis. The flow rate was set at 1.0 mL/min. 10μL of the sample was injected into the HPLC system for analysis and detected at 335 nm.

**Results**

The HPLC of the standard and SHHS Granules are shown in Figure 7 and Figure 8 respectively. There should be 10 characteristic peaks in the characteristic fingerprint of the test simple, among which the peak 8 should be consistent with the peak retention time of the corresponding reference substance, and the peak corresponding to the reference substance peak of nodakenin should be the S peak, and the relative retention time of each characteristic peak should be calculated, the relative retention time of peak 1 should be within ±15% of the specified value, and the relative retention time of the remaining peaks should be within ±10% of the specified value. The specified values are: 0.12 (peak 1), 0.27 (peak 2) , 0.36 (peak 3), 0.38 (peak 4), 0.41 (peak 5), 0.84 (peak 6), 0.92 (peak 7), 1.00 (peak 8), 1.19 (peak 9), 1.29 (peak 10).


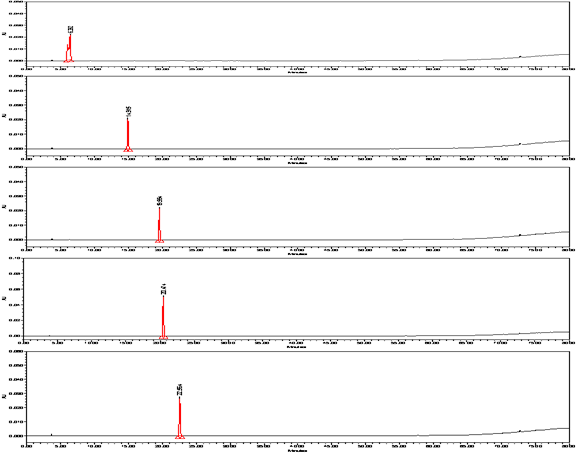


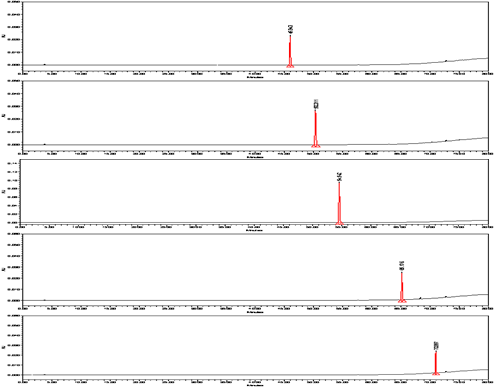


Figure 7. The HPLC profile of the control.


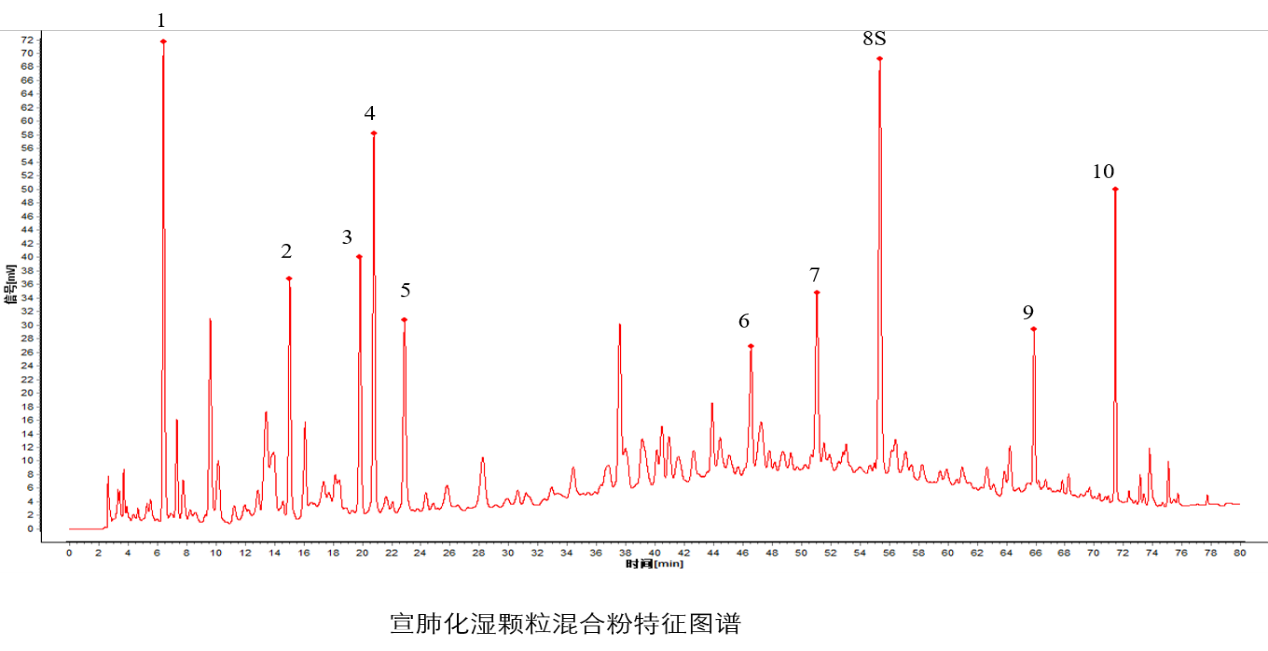
peak 1: adenine, peak 2: uridine, peak 3: guanosine, peak 4: inosine, peak 5: 5-hydroxymethyl-2-furaldehyde, peak 6: magnoloside A, peak 7: bergaptolo-β-glucoryranoside, peak 8 (S): nodakenin, peak 9: 6-Feruloylnodakenin, peak 10: 4-Hydroxyphenethylanisate

Figure 8: The HPLC chart of SHHS Granules

**Characteristic peak chemical composition mass spectrometry identification results**

| **Peak number** | **t_R_** | **ESI^+^** | **ESI^-^** | **Molecular weight**  **(measured value)** | **Molecular formula** | **Molecular weight**  **(theoretical value)** | **Molecular weight error (×10^-5^)** | **Compound** | **The main source of medicinal materials** |
| --- | --- | --- | --- | --- | --- | --- | --- | --- | --- |
| 1 | 7.52 | 136.0611 [M+H]^+^ | / | 135.0611 | C_5_H_5_N_5_ | 135.0545 | 4.9 | adenine | Mahuang、Kuxingren、Qianghuo、Tinglizi、Dilong、Xuchangqing、Mianmaguangzhong、Huoxiang、Cangzhu、Baizhu、Jiaoshenqu、Jiaomaiya |
| 2 | 14.40 | 245.0756 [M+H]^+^  113.0341[M-C_5_H_7_O_4_]^+^ | 243.0626 [M-H]-  487.1319 [2M-H]-^-^ | 244.0626 | C_9_H_12_N_2_O_6_ | 244.0695 | -2.8 | uridine | Qianghuo、Tinglizi、Dilong、Xuchangqing、guanghuoxiang、Peilan、Cangzhu、Baizhu |
| 3 | 20.79 | 284.0982 [M+H]^+^ | 282.0849 [M-H]^-^  565.1760 [2M-H]^-^ | 283.0849 | C_10_H_13_N_5_O_5_ | 283.0917 | -2.4 | guanosine | Qianghuo、Tinglizi、Dilong、Xuchangqing、Guanghuoxiang |
| 4 | 20.19 | 269.0874 [M+H]^+^  537.1673 [2M+H]^+^  137.0451 [M-C_5_H_7_O_4_]^+^ | 267.0742 [M-H]^-^  535.1549 [2M-H]^-^ | 268.0874 | C_10_H_12_N_4_O_5_ | 268.0808 | 2.5 | inosine | Dilong |
| 5 | 22.11 | 127.0386 [M+H]^+^ | / | 126.0386 | C_6_H_6_O_3_ | 126.0317 | 5.5 | 5-hydroxymethyl-2-furaldehyde | Jiaoshanzha、Jiaomaiya |
| 6 | 45.67 | 647.1939 [M+Na]^+^ | 623.2000 [M-H]^-^ | 624.2000 | C_29_H_36_O_15_ | 624.2054 | -0.9 | magnoloside A | Houpo |
| 7 | 50.16 | 365.0858 [M+H]^+^  387.0674 [M+Na]^+^  751.1459 [2M+Na]^+^  203.0331 [M-glc+H]^+^ | 423.0947[M+CH_3_COO]^-^  201.0199 [M-glc-H]^-^ | 364.0858 | C_17_H_16_O_9_ | 364.0794 | 1.8 | bergaptol-β-glucopyranoside | Qianghuo |
| 8 | 54.35 | 409.1500 [M+H]^+^  431.1308 [M+Na]^+^  247.0959 [M-glc+H]^+^  839.2731 [2M+Na]^+^ | 407.1359 [M-H]^-^  467.1578 [M+CH_3_COO]^-^  815.2793 [2M-H]^-^ | 408.1500 | C_20_H_24_O_9_ | 408.142 | 2.0 | nodakenin | Qianghuo |
| 9 | 64.74 | 607.1776 [M+Na]^+^， 1191.3659 [2M+Na]^+^ | 583.1839 [M-H]^-^ | 584.1839 | C_30_H_32_O_12_ | 584.18994 | -1.0 | 6-feruloylnodakenin | Qianghuo |
| 10 | 70.28 | 295.0933 [M+Na]^+^ | / | 272.0933 | C_16_H_16_O_4_ | 272.1049 | -4.3 | 4-hydroxyphenethylanisate | Qianghuo |
